# Efficacy and Safety of *P. hybridus* Leaf Extract Ze 339 for the Treatment of Allergic Rhinitis

**DOI:** 10.3390/arm93030013

**Published:** 2025-06-03

**Authors:** Verena M. Merk, Georg Boonen, Veronika Butterweck, Andreas Schapowal

**Affiliations:** 1 Medical Department, Max Zeller Söhne AG, 8590 Romanshorn, Switzerland; verena.merk@zellerag.ch (V.M.M.); georg.boonen@zellerag.ch (G.B.); 2 Schweizerische Medizinische Gesellschaft für Phytotherapie, 8021 Zurich, Switzerland

**Keywords:** *Petasites hybridus*, butterbur, Ze 339, safety, allergic rhinitis, hay fever

## Abstract

**Highlights:**

This review article focuses on safety and efficacy aspects of the *P. hybridus* leaf extract Ze 339 for the treatment of allergic rhinitis symptoms. By summarizing the available data from preclinical, clinical and non-interventional studies, as well as post-marketing pharmacovigilance monitoring, it provides a detailed overview of the therapeutic value of this herbal medicinal product.

**The main findings are as follows:**

- Ze 339 is efficient in the treatment of allergic rhinitis symptoms;
- Ze 339 is well-tolerated with a low incidence of adverse events.

**The implication of the main finding is the following:**

- Phytopharmaceuticals display an efficient and safe alternative to synthetic antihistamines in the treatment of allergic rhinitis.

**Abstract:**

Allergic rhinitis (AR) is a global health problem on the rise. More and more people are affected, and climate change is exacerbating this health problem in the long term. The quality of life of those affected is often severely compromised, and the financial burden on healthcare systems cannot be disregarded. Therefore, effective and safe medicines are needed to counteract this trend. *P. hybridus* (butterbur) leaf extract (Ze 339) displays a promising alternative to antihistamines in the treatment of AR symptoms. More than two decades after the first market launch it is now possible to draw a meaningful conclusion on its safety and efficacy. This review summarizes the available preclinical and clinical data, real-world data (RWD) as well as data from post-marketing pharmacovigilance monitoring about the herbal medicinal drug Ze 339. It focusses on the current knowledge about the mode of action as well as the evaluation of its efficacy and safety in the treatment of AR. Given its favourable safety profile and lack of sedative side effects, Ze 339 offers a valuable alternative to antihistamines and should therefore continue to be considered by medical practitioners for the treatment of allergic rhinitis symptoms.

## 1. Allergic Rhinitis: A Health Problem on the Rise

Allergic rhinitis (AR) is an immunoglobulin E (IgE)-mediated immune reaction to allergens, which leads to symptoms including itching in the nose, eyes or throat, sneezing and rhinorrhoea [1]. At a later stage, obstruction of the nose and sinuses may also occur [1]. A distinction is made between seasonal AR (pollen allergy, colloquially known as hay fever) and perennial AR (e.g., house dust mite allergy), with the severity of symptoms varying considerably from patient to patient [2]. In addition, AR not only has a negative effect on asthma control but is also part of the progressive atopy ‘atopic march’, and thus a risk factor for the development of asthma [3,4]. In general, AR is a widespread global health concern that can significantly reduce the quality of life of affected individuals [1].

The Robert Koch Institute (RKI, Berlin, Germany) has reported a marked increase in the prevalence of allergies (including AR) since the 1970s, with no indication of a reversal of this trend. One potential explanation for this phenomenon is climate change [5]. By the end of the century, the average annual temperature in the Alps and Alpine foothills is expected to rise by at least 1 °C to 4 °C, accompanied by increased precipitation [6]. This shift in climate is expected to have significant consequences for plant development and the pollen season [6]. Overall, it is expected, and in some cases already observed, that the earlier pollen season attributable to climate change and rising atmospheric carbon dioxide concentrations will result in an augmentation of pollen, an expansion of the pollen spectrum, and an increase in the allergenic potential of pollen [6]. In addition to climate-related factors, other environmental factors such as air pollution by industrial emissions appear to correlate with AR development [7]. Another highly recognized hypothesis about lifestyle-related risk factors for AR is the hygiene hypothesis. Already in 1989 David P. Strachan postulated the correlation between family hygiene standards and the development of atopic diseases [8]. According to this hypothesis, reduced exposure to microbial stimuli in early childhood, smaller family sizes and urban living may lead to an impaired development of the immune system and thus to an increasing susceptibility to atopic diseases [7]. Furthermore, the composition of the gut microbiome in infancy and its influence on the immune system also impacts the development of atopies including AR [7]. C-section, formula feeding and Western diet influence the gut microbiome and have been implicated with the development or exacerbation of allergic conditions [7]. Consequently, therapeutic interventions for AR are becoming increasingly important.

Avoidance of allergens is an obvious approach and can help to reduce symptoms [1,9]. Potential measures include the utilization of specialised bedding for house dust mite allergies or wearing masks for pollen allergies [9]. However, the complete avoidance of allergens is not a realistic option, and the effectiveness of these measures is not conclusively demonstrated by clinical data [9]. Additionally, it is important to ensure that any measures do not result in social isolation for those affected [1]. In instances where allergen avoidance measures are either insufficient or impractical, a range of medications are available. Antihistamines are a common treatment option. By antagonizing the histamine H1 receptors (H1R), they mitigate the symptoms triggered by the release of histamine in the acute phase of allergic reactions [1]. They can be administered orally, intranasally or ocularly, and are effective in both adults and children [1]. However, especially first generation antihistamines can cross the blood brain barrier and induce sedative effects [1]. Second and third generation antihistamines have fewer effects on the H1R in the brain and are therefore preferable [1]. In cases where antihistamines alone are insufficient, intranasal antihistamines can be administered concomitantly with intranasal corticosteroids. Corticosteroids are anti-inflammatory hormones that act through the glucocorticoid receptor (GR) and simultaneously induce the expression of anti-inflammatory cytokines and repress the expression of pro-inflammatory cytokines [10]. Corticosteroids are indicated for persistent or moderate to severe AR symptoms [10]. Although intranasal application shows reduced adverse effects compared to systemic administration, local irritation, dry nose and nasal bleeding may develop [10]. Specific immunotherapy (hyposensitization) is another option in the management of IgE-mediated AR [11]. By regular administration of standardized allergens in increasing doses over a period of several years, patients may become gradually tolerant to the specific allergen [11]. It has been shown that the reduction of allergen-specific T helper 2 cells, reduced production of allergen-specific IgE antibodies and the induction of regulatory T and B cells can ultimately alleviate the symptoms of AR [11]. In addition to the strategies for allergen avoidance, the use of antihistamines and corticosteroids, or specific immunotherapy, phytotherapeutics offer a promising therapeutic approach. Rational phytotherapy has its origins in folk medicine but is nowadays subject to strict quality controls and regulatory guidelines. Herbal medicines are only approved by authorities if their efficacy and safety are supported by clinical evidence. Approval is typically based either on monographed efficacy data from recognized sources (e.g., HMPC or ESCOP monographs) or on product-specific preclinical and clinical studies. The concept of rational phytotherapy is based exclusively on the utilization of scientifically tested medicinal products.

## 2. *P. hybridus* Leaf Extract Ze 339 as a Treatment Option for Allergic Rhinitis

**Figure 1:**
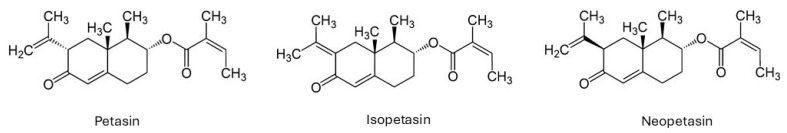
**Structural formulas of sesquiterpene esters neopetasin,** **petasin and isopetasin.**

*Petasites hybridus* (L.) P.G. Gaertn., B. Mey. & Scherb (Asteraceae) grows on moist, fertile soils, and often in proximity to rivers or streams [12]. While the use of rhizome extracts has a long history in folk medicine, with applications including migraine prophylaxis and the treatment of spastic pain, dysmenorrhea, coughs and wound healing [13,14], the subcritical CO_2_-extract Ze 339 prepared from the leaves of *P. hybridus* has been developed for the treatment of AR. Ze 339 is a lipophilic subcritical CO_2_ extract derived from the leaves of *Petasites hybridus*, and is a herbal medicinal product licensed in Switzerland and ten other countries for the treatment of AR [15]. It is indicated for the treatment of AR (hay fever) and its symptoms in the eyes, nose and throat [16]. Despite flavonoids and essential oils, the biologically active compounds in *P. hybridus* extracts are mainly the sesquiterpenes petasin, isopetasin and neopetasin which are found in the rhizomes and leaves of the plant (Figure 1) [13,14].

In addition to therapeutically beneficial substances, *Petasites* species contain toxicologically relevant pyrrolizidine alkaloids (PAs) and their N-oxide derivatives [17]. From a biological standpoint, these substances represent critical defence mechanisms to protect the plant from herbivores, but for medical applications, they need to be kept to a minimum [17]. It is therefore critical to select suitable plant varieties and production processes to minimize the concentration of PAs in the extracts and to confirm this by routine analytical methods. Schenk et al. 2015 developed an ultra-high performance liquid chromatography-time-of-flight mass spectrometry (UPLC-TOF-MS) method to quantify several PAs in Ze 339 [18]. This method appeared to be highly sensitive with a limit of quantification for all PAs at 2 ppm enabling the detection of trace concentrations [18]. Analyses of Ze 339 by this method confirmed the effective removal of PAs in the CO_2_-extraction process. To further rule out possible hepatotoxicity and to assess the safety profile, the extract was tested in different in vitro models [19]. Metabolic activity upon Ze 339 treatment has been assessed in two human (HepG2, HepaRG) and one rodent (H-4-II-E) cell lines using the water-soluble-Tetrazolium-1 (WST-1) test [19]. The human cell lines displayed a mild to moderate decrease in metabolic activity relative to the control at the highest concentration of 100 μg/mL [19]. Metabolic assays with S9 fractions from human, rat and dog liver homogenates further revealed a species-dependent metabolic activity mediated by cytochrome P 450 enzymes. Human S9 fractions showed the lowest metabolic activity towards petasin isomers [19]. These findings indicate that Ze 339 is only toxic in supraphysiological doses, suggesting a safe application for medical purposes [18,19]. However, this alone is not sufficient to ensure safe and effective use by patients. Nowadays, herbal pharmaceuticals are subject to rigorous safety and efficacy requirements. Over the past two decades, a substantial amount of additional preclinical and clinical evidence has been generated to substantiate the efficacy and safety of Ze 339 but also to reveal its mode of action.

## 3. Understanding the Mode of Action of Ze 339

The general course of immune responses that culminate in the development of AR is similar across the diverse allergens. During the sensitization phase, allergens are recognized and processed by dendritic cells which leads through the activation of CD4+ T cells to the production of allergen-specific IgE antibodies by plasma cells [1]. IgE antibodies are distributed through the circulation and bind to high affinity IgE receptors on effector cells (e.g., mast cells, basophilic granulocytes) [1]. Upon re-exposure, the allergens directly bind to the specific IgE antibodies on mast cells resulting in the release of pre-stored allergic mediators (e.g., histamine, leukotrienes) [1]. The subsequent stimulation of sensory nerves and nasal vasculature immediately leads to the induction of typical acute symptoms of AR [1]. Acute symptoms are accompanied by inflammatory symptoms, such as nasal obstruction initiated by vasodilation and infiltrating immune cells. These are induced by newly synthesised pro-inflammatory cytokines and chemokines. The detailed pathophysiology of AR has been reviewed extensively before [1].

**Figure 2:**
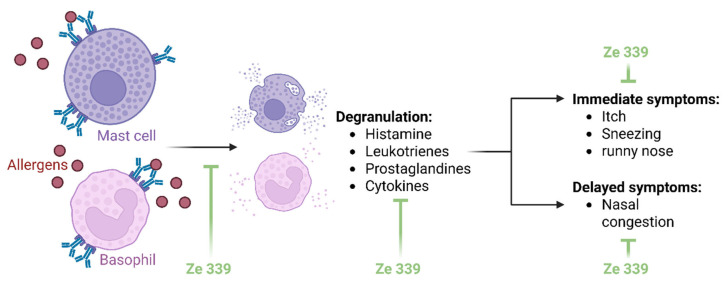
**Schematic overview of the hypothesized mode of action of Ze 339.** Ze 339 inhibits the synthesis and release of allergic mediators from allergen-stimulated mast cells and basophils. This relieves both immediate and delayed symptoms associated with inflammation. The figure was created with Biorender.com.

Elucidating the mechanisms of action of herbal extracts is very complex. Extracts are mixtures of several substances whose effects cannot be attributed to a single mechanism. Nevertheless, the mechanisms of action of *P. hybridus* extract Ze 339 are now comparatively well understood. Ze 339 appears to alleviate AR symptoms by intervening the allergic reaction on two levels (Figure 2): firstly, by inhibiting acute phase symptoms such as rhinorrhoea and secondly, by reducing late phase symptoms such as nasal obstruction [20,21].

**Figure 3:**
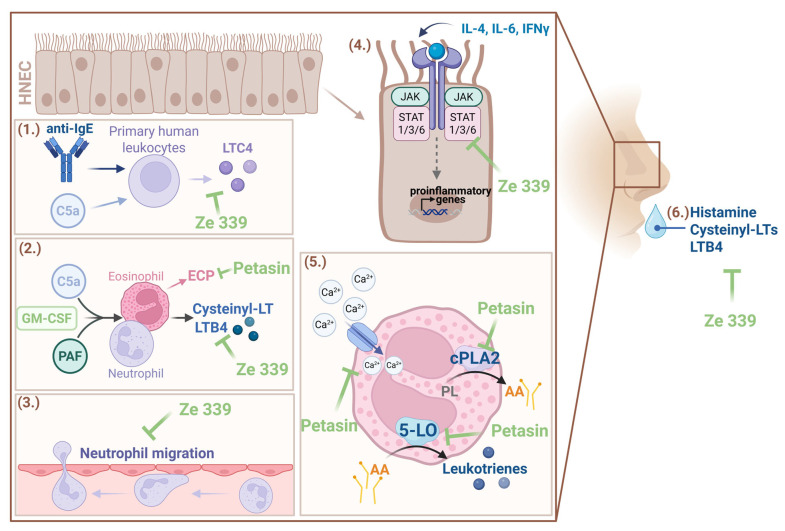
**Schematic overview of preclinical data from Ze 339.** (**1.**) Treatment of primary human leukocytes with Ze 339 led to decreased anti-IgE or complement C5a-stimulated LTC4 synthesis. (**2.**) GM-CSF-priming and complement C5a or PAF stimulation of eosinophils and neutrophils led to the synthesis of cysteinyl LTs and LTB4 which was inhibited by Ze 339. Furthermore, treatment of stimulated eosinophils with petasin reduced the release of ECP. (**3.**) The treatment with Ze 339 resulted in decreased neutrophil migration towards HNEC supernatant. (**4.**) Ze 339 inhibited cytokine-induced JAK/STAT signalling. (**5.**) Treatment of stimulated eosinophils with petasin resulted in reduced intracellular Ca^2+^, reduced cPLA2 activity and reduced 5-LO activity. (**6.**) Ze 339 application reduced histamine, cysteinyl LTs and LTB4 concentration in nasal fluids of patients. Abbreviations: AA: Arachidonic acid; anti-IgE: anti-immunoglobulin E antibody; complement C5a: complement component 5a; cPLA2: cytosolic phospholipase A2; cysteinyl LTs: cysteinyl leukotrienes; ECP: eosinophil cationic protein; GM-CSF: granulocyte–macrophage colony-stimulating factor; HNEC: human nasal epithelial cells; IL: interleukin; JAK: Janus kinases; LTB4: leukotriene B4; LTC4: leukotriene C4; PAF: platelet activating factor; PL: phospholipids; STAT: signal-transducer and activator of transcription protein; 5-LO: 5-lipoxygenase. The figure was created with Biorender.com.

As early as 1998, preclinical research provided initial indications of the anti-allergic mode of action of *P. hybridus* extracts [22]. Experiments with primary human peripheral leukocytes revealed that petasin-containing extracts of *P. hybridus* effectively inhibited cysteinyl leukotrienes (i.e., LTC4) synthesis and release from complement C5a or anti-IgE-receptor antibody-stimulated immune cells (Figure 3) [22]. Subsequent investigations by Thomet et al. have enabled more detailed understanding of the principle of action [20,21,23,24]. In vitro analyses revealed that LTB4 synthesis by neutrophils and cysteinyl leukotrienes synthesis by eosinophils were dose-dependently decreased by Ze 339 treatment [20]. LTB4 has been shown to stimulate superoxide generation while cysteine-leukotrienes increase microvascular permeability [20]. Therefore, Ze 339 limits the inflammatory downstream effects of leukotrienes. Moreover, Ze 339 efficiently blocked platelet-activating factor (PAF) and complement C5a induced increases in intracellular calcium [20]. This could limit the activation of calcium-dependent enzymes such as cytosolic phospholipase A2 (cPLA2) and 5-lipoxygenase (5-LO) which are required for leukotriene synthesis [20].

By analyzing the effects of isolated *P. hybridus* sesquiterpenes on eosinophils and neutrophils, similar effects were observed as for the total extract [21]. While petasin, isopetasin and neopetasin showed comparable and potent inhibitory effects on cysteinyl-leukotriene synthesis, their effects on eosinophil cationic protein (ECP) release, 5-LO translocation to the nuclear membrane and cPLA2 enzymatic activity appeared to be differential (Figure 3) [21]. These studies indicate that Ze 339 and petasins inhibit the signalling pathway at or prior to the level of cPLA2 and 5-LO activation (Figure 3) [20,21]. Moreover, it has been shown that the inhibition of leukotriene synthesis is dependent on the sum of petasins [25]. A small open trial with six patients suffering from AR revealed a significant reduction of histamine, cysteinyl LTs and LTB4 in nasal fluids after five days of Ze 339 treatment [24] (Figure 3). Moreover, while immunophenotyping analyses revealed no changes in leukocyte numbers, rhinomanometric flow, nasal congestion and itching, rhinorrhoea, sneezing as well as quality of life improved significantly [24]. Initial pilot studies in humans have provided further insight into the mechanism of action. A small randomized controlled trial (RCT) in 8 patients with respiratory allergy and 10 healthy volunteers excluded a direct antihistaminic effect of Ze 339 [26]. Ze 339 had no effect on skin test reactivity using histamine, codein, methacholine and aeroallergen solutions [26]. This suggests a mode of action for Ze 339 that does not act like synthetic antihistamine through the histamine receptor. This has the advantage that sedative side effects via HR1 in the central nervous system (CNS) are unlikely. Interestingly, there have been reports of patients who have taken Ze 339 and experienced an improvement in food-related histamine intolerance (HIT) [27]. HIT is characterized by various symptoms in response to exogenous histamine that resemble an allergic reaction with effects on the intestine, the respiratory system, the cardiovascular system, the nervous system and the skin [28]. This is caused, among others, by insufficient elimination of histamine by intestinal diamine oxidase (DAO) [28]. An *in vitro* study that investigated the effect of Ze 339 on HIT revealed no influence of Ze 339 on DAO protein expression and enzyme activity [27]. However, Ze 339 decreased the organic cation transporter 3 (OCT3)-mediated cellular accumulation of histamine which may contribute to improved symptoms in patients suffering from HIT [27]. Nevertheless, the efficacy of Ze 339 in this indication remains to be substantiated.

More detailed investigations into the involved molecular signalling pathways have been carried out by Steiert et al. 2017 [29]. As symptom patterns in response to allergic reactions and various infections are at least partially similar, this study investigated the immunomodulatory effect of Ze 339 under pattern-associated molecular pattern (PAMP) (bacterial mimetics), poly I:C (viral mimetic) and cytokine-induced inflammatory conditions in primary human nasal epithelial cells (HNEC) [29]. Whereas Ze 339 did not alter the cytokine and chemokine response upon stimulation with bacterial mimetics, the study showed decreased neutrophil migration and decreased cytokine and chemokine production in poly I:C treated cells [29]. Interestingly, Ze 339 modulated the inflammatory response in HNECs treated with interleukin (IL)-4, IFN-γ and IL-6. This was most likely due to an inhibition of the Janus kinases (JAK), a signal transducer and activator of transcription proteins (STAT) pathway by Ze 339, as shown in Western blot and immunofluorescence analyses, especially for IL-6 treatment [29]. As the petasins in Ze 339 are highly hydrophobic, the authors hypothesize that they could alter JAK/STAT complex assembly by accumulating in the plasma membranes [29] (Figure 3). However, the exact mechanism is up to further investigations.

In conclusion, the current knowledge on the mode of action can be summarized as follows: During an allergic reaction, Ze 339 reduces early phase symptoms potentially via the inhibition of histamine and leukotriene release from effector cells rather than direct antihistaminic effects and alleviates late-phase symptoms such as nasal obstruction through the inhibition of leukotriene synthesis. Additionally, pro-inflammatory cytokine production in response to allergens is inhibited potentially via interference of the JAK/STAT signalling pathway (Figure 3).

## 4. Clinical Evidence for the Use of Ze 339: Anti-Allergic and Anti-Inflammatory Effects

Four RCTs with a total of 659 patients with AR have been performed to prove the anti-allergic and anti-inflammatory effects of Ze 339. The first clinical trial has been conducted by Schapowal et al. in 2002 [30]. This randomized, double-blind, parallel group, multicentric study with 125 patients suffering from seasonal AR assessed the effects and safety of Ze 339 compared to the synthetic antihistamine cetirizine [30]. After two weeks of treatment, the clinical global impression (CGI) score was determined using a validated questionnaire (SF-36) [30]. Not only were the improvements similar in both treatment groups, but also the incidence of adverse events were similar (Ze 339: 10 adverse events, cetirizine: 12 adverse events) [30]. Importantly, while Ze 339 had no sedative effects, cetirizine caused drowsiness and fatigue in some patients [30].

In 2004, the first placebo-controlled clinical trial was conducted [31]. High and low doses of Ze 339 were tested for efficacy and safety in comparison to a placebo over a course of two weeks [31]. The primary endpoint of this study was the change in symptoms measured by visual analogue scale (VAS) while CGI score and responder rate were defined as secondary endpoints [31]. Ze 339 showed a dose-dependent effect in relief of AR symptoms and was significantly more effective than the placebo regarding symptom reduction and CGI score [31]. Only mild and primarily gastrointestinal adverse events were documented and the incidence was found to be low and comparable within the treatment groups [31]. It should also be emphasized here that fatigue was not a reported side effect, suggesting that Ze 339 displays an alternative for antihistamines with sedative adverse effects [31].

Another randomized placebo-controlled parallel-group study investigated the efficiency and safety of Ze 339 compared to the antihistamine fexofenadine [32]. A total of 330 patients with intermittent AR were treated for 2 weeks [32]. The total symptom score (TSS) from the beginning to the end of the study was defined as the primary endpoint. Secondary endpoints included the instantaneous TSS (assessed just before the next dose), the combined TSS (including nasal congestion), the physician’s global assessment of symptoms and the responder rate [32]. Ze 339 and fexofenadine showed comparable efficiency and were both significantly more effective than the placebo. This study confirmed the good safety profile of Ze 339 observed in previous trials [32]. The incidence of adverse events was comparable in all treatment groups and there were no changes in the assessed safety parameters (liver function tests, vital signs, electrocardiogram (ECG)) [32]. Liver function tests included determination of serum glutamate-oxalacetate aminotransferase (SGOT) (U/L), serum glutamate-pyruvate-transaminase (SGPT) (U/L) and gamma-glutamyltransferase (γ-GT) (U/L) values at baseline and at the end of the study [32]. Consistent with previous findings, adverse events related to sedation were predominantly reported in the group receiving antihistamines [32].

Finally, Dumitru et al. 2011 conducted a prospective, randomized, double-blind, double-dummy, 3-arm crossover study with 18 adult volunteers with at least a 2-year medical history of moderate-to-severe AR to grass pollen [33]. The primary objective was to assess the resolution of nasal obstruction upon allergen challenge [33]. In addition, nasal secretions were collected for analysis of inflammatory mediators, and the global assessment of the nasal condition was conducted by patients using a VAS, with scores ranging from 0 to 10 [33]. Study participants received Ze 339, desloratadine or a placebo for five days in different treatment sequences. To assess the impact of Ze 339, desloratadine and the placebo, they performed rhinomanometry and local inflammatory cytokine measurements 24 h after the allergen challenge [33]. The time to recover the nasal airflow was reduced by Ze 339 to 5.4 h compared to 10.7 h for desloratadine and 9.1 h for the placebo [33]. Moreover, IL-8 and leukotriene B4 levels in nasal secretion were reduced by Ze 339 suggesting an anti-inflammatory effect [33]. During the study, no serious side effects were documented and safety profiles of all three treatments were similar [33]. Six adverse events (headache, dysgeusia, urticaria, procedural pain, head pressure and nosebleed) occurred in the Ze 339 group, five adverse events (fatigue, dizziness, tiredness, nausea and sneezing) occurred with desloratadine and eight adverse events occurred with the placebo (vomiting, loose stools, toothache, nose swelling, dizziness, nausea, headache and Hashimoto thyroiditis (assessed as being preexisting)) [33]. Despite the low number of study participants and the comparatively shorter treatment duration, this study clearly demonstrates the efficacy and safety of Ze 339 with precise measurement methods.

All studies concluded that Ze 339 is an effective and well-tolerated treatment for AR symptoms and a good alternative to common antihistamines. From the conducted clinical trials, there is no evidence that Ze 339 exhibits the sedative effects commonly associated with typical antihistamines. It should also be emphasised that Ze 339 not only effectively inhibits the immediate symptoms but also the inflammation at later stages of the allergic reaction.

## 5. Real-World Data Confirm the Safety and Efficacy of *P. hybridus* Leaf Extract Ze 339

Real-world data (RWD) are obtained from routine post-marketing surveillance [34]. RWD are important for pharmacovigilance and form the basis of real-world evidence (RWE), i.e., the evidence that confirms the safety and efficacy of a medical product in general practice [34]. While RWE is unable to replace RCTs, it offers invaluable insights into the actual use of drugs. As patients are usually not excluded due to comedication or concomitant diseases, these studies give a ‘real’ picture of the general population and allow conclusions about potential drug interactions. Between 2004 and 2021, four studies were published that analyzed RWD on the use of Ze 339 [35,36,37,38]. A total of 1874 patients were included in these observational studies, and a favourable safety and effectiveness profile was observed [35,36,37,38]. Most patients were recruited during the peak hay fever season to enable the investigation of the efficacy and safety of Ze 339 on pronounced AR symptoms. All studies evaluated typical AR symptoms such as rhinorrhoea, nasal congestion, sneezing, itchy nose and eyes and red eyes before, during and after the treatment with Ze 339 [35,36,37,38].

Keusch at al. 2004 conducted an open, prospective post-marketing surveillance study including 141 patients, which was carried out by 38 medical doctors in Switzerland [35]. The use of Ze 339 was in general very well tolerated with only mild adverse effects in nine patients. During the observation period of two weeks, patients reported cases of gastrointestinal complaints (mild nausea, flatulence and stomach cramps), but also occasional tiredness, pruritus and muscle cramps. However, no serious adverse events occurred [35]. Moreover, the presence of comorbidities such as asthma, allergies and skin diseases or risk factors such as pets and smoking did not impact the efficacy of Ze 339 [35]. In conclusion, the collected RWD indicates that Ze 339 leads to fast and efficient AR-symptom reduction with a good tolerability and safety profile [35].

Käufeler et al. 2006 analyzed data from an open, non-interventional post-marketing surveillance study including 580 patients with seasonal AR who took Ze 339 for two weeks [36]. In total, 92% of the patients positively rated the tolerability with only 3.8% of patients experiencing mild adverse events mostly related to gastrointestinal complaints. Similarly to the study by Keusch et al., no serious adverse effects related to the study medication occurred. A total of 56.7% of patients exclusively received Ze 339, while 43.3% received one or two comedications (such as nasal sprays, antihistamines, eyedrops) [36]. Comedication did not result in greater efficiency than Ze 339 alone. The authors highlighted fewer sedative effects of Ze 339 compared to antihistaminic drugs [36].

The post-marketing surveillance study by De Marquis et al. 2012 included 927 patients in South America not only with seasonal but also with perennial AR [38]. The tolerability was rated as overall favourable by physicians (90.7%) as well as by patients (94.47%) with no severe adverse events during the treatment period of 28 days [38]. In this study, 63.6% of patients used Ze 339 alone, while 36.4% combined it with other antiallergic medications, mostly antihistamines. No relevant interactions were reported [38]. It can be concluded that Ze 339 is not only safe and efficient in seasonal AR but also in perennial AR [38].

Blosa et al. 2021 analyzed another non-interventional, observational study conducted by 62 general practitioners and medical specialists (allergologists) in Switzerland [37]. The safety profile was favourable, with only three patients reporting a total of four mild adverse events that were related to the gastrointestinal system [37]. These were nausea, malaise and abdominal pain and did not require treatment [37]. No serious adverse events were recorded [37]. Moreover, the observed application periods in this study enabled the analysis of long-term effects [37]. The average treatment duration was 63 days and 75% of the patients were treated for at least four weeks [37]. Long-term use did not lead to an increased incidence of side effects and 67 patients continued the therapy beyond the study [37]. The collected RWD for Ze 339 further allowed for conclusions about potential drug interactions. Overall, 58.5% of patients were treated exclusively with Ze 339, while 41.5% received combination therapy with other allergy medications including antihistamines, sympathomimetics or leukotriene antagonists [37]. No relevant drug interactions were reported for patients receiving combination therapy with either substance [37]. In conclusion, this study showed the high efficacy of Ze 339 and it did not reveal safety concerns or signs of tolerance which makes it suitable for long-term use [37]. Interestingly, patients also reported significant relief of other atopies such as atopic dermatitis and allergic asthma, indicating that Ze 339 may also have potential for the treatment of other atopic diseases [37].

Although AR is not a life-threatening condition, it can have a significant impact on the quality of life (QoL) of those affected. All four observational studies included QoL parameters in their real-world data collection. The evaluation was based on a subjective assessment by the patients and their treating physicians. The assessment covered factors, including daytime tiredness, difficulties concentrating and impairment of daily activities. All studies show a substantial improvement in QoL due to a reduction in allergic symptoms. Patients felt less restricted in their daily lives and rated the overall efficacy as positive. This is supported by the fact that two of the four observational studies reported that many patients (Keusch et al. 2004: 104 patients, Blosa et al. 2021: 67 patients) continued treatment beyond the observation period [35,37]. Although this is an important finding, the other two studies did not take this into account during their collection of data [36,38].

A critical aspect that also contributes to the effectiveness of a drug is compliance [39]. Medication compliance refers to how well a patient follows the prescribed dosage and schedule [40]. Medication nonadherence could thus lead to the worsening of symptoms and the overall condition and should be avoided [39]. It is therefore important to understand how patients are adhering to a particular medication in order to make any necessary adjustments or develop strategies to improve compliance. RWD provides a more accurate assessment of patient compliance within routine clinical settings compared to the controlled environments of randomized controlled trials (RCTs). All four observational studies included medication compliance to Ze 339 in their datasets and drew a positive conclusion. Three of the four studies reported that the majority of patients adhered to the recommended dosage [35,36,37]. However, precise numerical data are only provided by De Marquis et al. 2012 [38]. Overall, 83.5% of patients continued to take Ze 339 until the end of the study [38].

Herbal medicinal products containing *P. hybridus* leaf native extract Ze 339 were first authorized in Switzerland in 2003 and are now authorized in ten different countries. The analyses of the periodic safety update report (PSUR) for Ze 339, considering all pharmacovigilance data since market authorization including all doses sold, showed a ratio of one adverse reaction per 469500 defined daily doses (company internal data). These data further confirm the excellent safety profile for this herbal drug.

## 6. Future Therapeutic Potential: Antiviral Activity of Ze 339

Interestingly, some key symptoms of seasonal allergies, such as coughing, fatigue, headaches or congestion, are also characteristic of other medical conditions, such as viral infections. In this context, the latest preclinical research is of particular importance, as it may open a new field of application for Ze 339. Independently of its influence on anti-allergic immune reactions, Ze 339 also appears to have antiviral activity against specific virus strains. The first indications for antiviral activity can be found in the study of Steiert et al. 2017 [29]. It was demonstrated that Ze 339 reduces immune responses in human nasal epithelial cells triggered by diverse stimuli including poly I:C, a synthetic analogue of double-stranded RNA that simulates viral infections by activating Toll-like receptor 3 (TLR3) [29].

In the following, two studies investigated the antiviral activity of Ze 339 in more detail. Urda et al. 2022 [41] and Jakwerth et al. 2023 [42] both show an antiviral effect of Ze 339 against SARS-CoV-2 in different cell culture models. Urda et al. (2022) performed virus infection assays in cell lines with epithelial morphology (Vero E6 and Calu-3 cells) using Wuhan and Delta variants of SARS-CoV-2 [41]. They found a dose-dependent inhibition of viral infection by Ze 339 in plaque assays and via RT-qPCR measurements of viral RNA levels [41]. Comparison of the IC50 values of remdesivir (1.53–2.37 µM), a nukleotide analogue, and Ze 339 (0.1–0.4 µg/mL) suggested strong antiviral potency for Ze 339, which was even more potent than remdesivir [41]. Jakwerth et al. 2023 performed their analyses in normal human bronchial epithelial cells (NHBE) for a more physiologically relevant model [42]. They proved antiviral activity of Ze 339 also against the Omicron variant of SARS-CoV2 in plaque assays and RT-qPCR analyses [42]. Furthermore, to visualize and track the viral spread, they used a GFP-expressing SARS-CoV-2 infection model and live cell imaging [42]. The results showed a significant reduction of GFP signal in the presence of Ze 339 [42]. In addition, RNA sequencing was performed to investigate potential immune modulation. Indeed, Ze 339 reduced the expression of several interferon genes, especially of IFNA10 [42].

Collectively, Ze 339 displays a promising antiviral candidate against SARS-CoV-2 but potentially also against other respiratory viruses that exploit similar cellular mechanisms. However, this needs to be confirmed in further preclinical and clinical studies. A notable advantage of Ze 339 is its pre-existing authorization as a medicinal product, and its favourable safety profile. Consequently, the utilization of Ze 339 as an antiviral agent would fall under the paradigm of drug repurposing. Therefore, it has the potential to decrease patient risk and development expenses, as well as shortening authorization times for novel treatment options [43].

## 7. Conclusions

**Figure 4:**
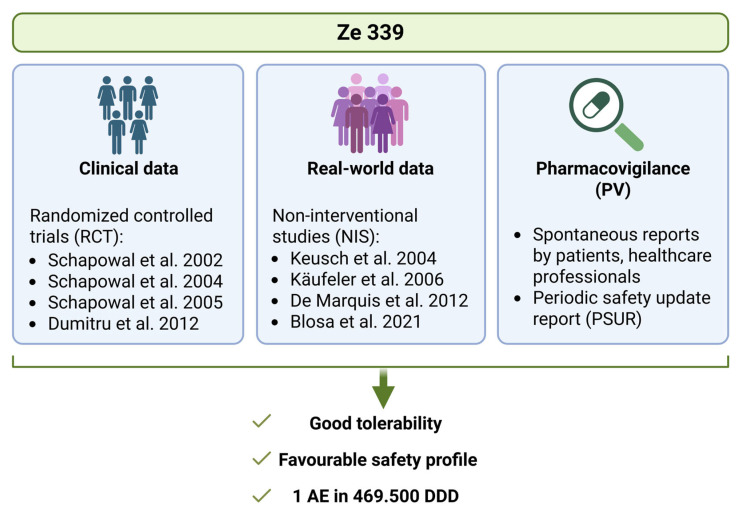
**Overview of efficacy and safety data of Ze 339**. List of published randomized controlled trials (RCT) and non-interventional studies (NIS) [30,31,32,33,35,36,37,38]. Abbreviations: AE: adverse event, DDD: defined daily doses. The figure was created with Biorender.com.

Since the marketing authorization of Ze 339 in 2003 for the treatment of AR symptoms, the drug has appeared to be safe and well tolerated with only mild gastrointestinal side effects in a small percentage of patients. In none of the conducted studies did any serious side effect related to the study drug occur, and the PSUR confirms a low incidence of side effects in general (Figure 4).

In the future, further efforts should be made to fully understand the mechanism of action and also to evaluate the potential for antiviral treatment in clinical trials. Currently, Ze 339 is authorized in ten countries for the following indication: treatment of symptoms of allergic rhinitis (hay fever) as well as related symptoms in eyes, nose and throat.

Overall, Ze 339 appears as a safe alternative to antihistamines with no sedative effects. The efficacy is comparable to that of other medications in this indication, and thus the evidence justifies the routine use in the treatment of AR symptoms.

## Data Availability

Data sharing is not applicable.

## Abbreviations

The following abbreviations are used in this manuscript:

AA	Arachidonic acid
5-LO	5-lipoxygenase
anti-IgE	Anti-immunoglobulin E antibody
AR	Allergic rhinitis
CGI	Clinical global impression
CNS	Central nervous system
complement C5a	Complement component 5a
cPLA2	Cytosolic phospholipase A2
cysteinyl-LTs	Cysteinyl leukotrienes
DAO	Diamine oxidase
ECG	Electrocardiogram
ECP	Eosinophil cationic protein
EMA	European Medicines Agency
GM-CSF	Granulocyte–macrophage colony-stimulating factor
GR	Glucocorticoid receptor
H1R	Histamine H1 receptor
HIT	Food-related histamine intolerance
HNEC	Human nasal epithelial cells
IFN	Interferon
IgE	Immunoglobulin E
IL	Interleukin
JAK	Janus kinases
LTB4	Leukotriene B4
LTC	Cysteinyl-leukotrienes
LTC4	Leukotriene C4
LTC4	Leukotrienes C4
NHBE	Normal human bronchial epithelial cells
OCT3	Organic cation transporter 3
PA	Pyrrolizidine alkaloids
PAF	Platelet activating factor
PAMP	Pattern-associated molecular pattern
PL	Phospholipids
PSUR	Periodic safety update report
QoL	Quality of life
RCT	Randomized control trial
RKI	Robert Koch Institute
RWD	Real world data
RWE	Real world evidence
SGOT	Serum glutamate-oxalacetate aminotransferase
SGPT	Serum glutamate-pyruvate-transaminase
STAT	Signal-transducer and activator of transcription protein
TLR3	Toll-like receptor 3
TSS	Total symptom score
UPLC-TOF-MS	Ultra-high performance liquid chromatography-time-of-flight mass spectrometry
VAS	Visual analogue scale
WST-1	Water-soluble-Tetrazolium-1
γ-GT	Gamma-glutamyltransferase
